# Technology Acceptance and Usability of a Mobile App to Support the Workflow of Health Care Aides Who Provide Services to Older Adults: Pilot Mixed Methods Study

**DOI:** 10.2196/37521

**Published:** 2022-05-18

**Authors:** Antonio Miguel Cruz, Hector Perez Lopez Portillo, Christine Daum, Emily Rutledge, Sharla King, Lili Liu

**Affiliations:** 1 Glenrose Rehabilitation Research, Innovation & Technology Glenrose Rehabilitation Hospital Edmoton, AB Canada; 2 Department of Occupational Therapy Faculty of Rehabilitation Medicine University of Alberta Edmonton, AB Canada; 3 Faculty of Health University of Waterloo Waterloo, ON Canada; 4 Educational Psychology Faculty of Education Edmonton, AB Canada

**Keywords:** usability, technology acceptance, Unified Theory of Acceptance and Use of Technology, UTAUT, older adults, caregivers, health care aides, mobile phone

## Abstract

**Background:**

Health care aides are unlicensed support personnel who provide direct care, personal assistance, and support to people with health conditions. The shortage of health care aides has been attributed to recruitment challenges, high turnover, an aging population, the COVID-19 pandemic, and low retention rates. Mobile apps are among the many information communication technologies that are paving the way for eHealth solutions to help address this workforce shortage by enhancing the workflow of health care aides. In collaboration with Clinisys EMR Inc, we developed a mobile app (Mobile Smart Care System [mSCS]) to support the workflow of health care aides who provide services to older adult residents of a long-term care facility.

**Objective:**

The purpose of this study was to investigate the technology acceptance and usability of a mobile app in a real-world environment, while it is used by health care aides who provide services to older adults.

**Methods:**

This pilot study used a mixed methods design: sequential mixed methods (QUANTITATIVE, qualitative). Our study included a pre– and post–paper-based questionnaire with no control group (*QUAN*). Toward the end of the study, 2 focus groups were conducted with a subsample of health care aides (*qual*, qualitative description design). Technology acceptance and usability questionnaires used a 5-point Likert scale ranging from *disagree* (1) to *agree* (5). The items included in the questionnaires were validated in earlier research as having high levels of internal consistency for the Unified Theory of Acceptance and Use of Technology constructs. A total of 60 health care aides who provided services to older adults as part of their routine caseloads used the mobile app for 1 month. Comparisons of the Unified Theory of Acceptance and Use of Technology constructs’ summative scores at pretest and posttest were calculated using a paired *t* test (2-tailed). We used the partial least squares structural regression model to determine the factors influencing mobile app acceptance and usability for health care aides. The α level of significance for all tests was set at *P*≤.05 (2-tailed).

**Results:**

We found that acceptance of the mSCS was high among health care aides, performance expectancy construct was the strongest predictor of intention to use the mSCS, intention to use the mSCS predicted usage behavior. The qualitative data support the quantitative findings and showed health care aides’ strong belief that the mSCS was useful, portable, and reliable, although there were still opportunities for improvement, especially with regard to the mSCS user interface.

**Conclusions:**

Overall, these results support the assertion that mSCS technology acceptance and usability are high among health care aides. In other words, health care aides perceived that the mSCS assisted them in addressing their workflow issues.

## Introduction

### Background

Health care aides are unlicensed support personnel who provide direct care, personal assistance, and support to people with health conditions that affect their daily function [1]. Currently, there is a shortage of health care aides due to challenges in recruitment, high turnover, an aging population, the COVID-19 pandemic, and low retention rates. For example, a recent study conducted in the United States found that the 3-year retention rates among health care aides were as low as 36% [2]. In Canada, there is a shortage of health care aides who provide care to older adults. As a result, Canada is seeking approximately 200,000 new health care aides over the next 10 years to meet the needs of the growing aging population [3].

Workflow issues have a negative impact on health care aides’ job satisfaction and quality of care. The scope of practice and decision-making, service authorization and access to client information, relationships, safety, critical incidents, communications, documentation, travel, scheduling and navigation, and education are the most common workflow issues identified by health care aides [4]. The implementation of information communication technologies (ICTs) can improve workflow issues and job satisfaction [4]. There is a positive correlation between job satisfaction and employee retention [5]. Multifeatured mobile apps are among the many ICTs paving the way for eHealth solutions in workforce shortages [6,7]. With various modes of implementation, such as telemonitoring and electronic health records, the development of ICTs has the potential to benefit workflow and tasks within the health care sector [8,9].

Knowledge of the usability and acceptance ICTs in health care settings is imperative for the success of ICT deployment. Perez et al [10] recently identified the drawbacks and benefits of ICT adoption by health care aides. A major deterrent is the cumbersome and time-consuming nature of the adoption and implementation of ICTs. In contrast, the major benefits include improved workflow, inclusion of time management skills, protocol simplification, standardized procedures, and staff scheduling. Moreover, the lack of ICT solutions for care providers of persons living with dementia is highlighted by Grossman et al [11]. This study identified >200,000 mobile health (mHealth) apps, only 22 of which were intended for dementia care. To reduce the burden on caregivers, the literature identifies useful ICT features, including information and resources, family communication and coordination, memory aids for care activities and socialization, carer support resources, medication management, and personal health records [11].

The COVID-19 pandemic has been a major contributor to the recent uptake of technology [12]. A study analyzing older adults’ experiences using technologies reported that more than half of the participants had adopted new technologies since the beginning of the pandemic [13]. In clinical settings, real-time health information has become a key feature of ICT solutions, owing to the infectious nature of the virus. The inherent need for modernized technology deployment in long-term care settings during COVID-19 outbreaks and in a postpandemic world is critical for support staff such as health care aides [12].

In light of the COVID-19 pandemic, an understanding of user-technology interactions is fundamental to ICT design and deployment in care settings. Health care aides, nurse managers, and other health care providers benefit from ICT use through improved communication, workflow support, and information accessibility [10,14]. Furthermore, there is an opportunity to enhance communication between clients and their family members [15]. The impact of this understanding can improve workflow issues, job satisfaction, and job retention in health care aides.

In collaboration with Clinisys EMR Inc, we developed a mobile app intended to support the workflow of health care aides who provide services to the older adult residents of a care facility. The mobile app was trialed in a long-term care setting by health care aides. Thus, the purpose of this study was to investigate the technology acceptance and usability of a mobile app in a real-world environment, while it is used by health care aides who provide services to older adult residents.

### Theoretical Framework: Brief Description

Technology acceptance relates to user beliefs, whereas usability is a concept associated with the actual use of technology [16]. Theories that explain the acceptance and adoption of technologies are based on 2 foundational theories that posit why an individual chooses whether to use a technology. These theories are the Theory of Planned Behavior [17] and its predecessor, the Theory of Reasoned Action [18], which are based on the main premise: as much of human behavior is under volitional control, most behaviors can be accurately predicted from an *appropriate measure* of the individual’s intention to perform the behavior in question [19]. The Unified Theory of Acceptance and Use of Technology (UTAUT), in its UTAUT [20] and UTAUT2 [21] versions, has emerged as the dominant model explaining the behavioral intention to use technologies and behavior connected to the use of technologies. The UTAUT posits that performance expectancy, effort expectancy, and social influence are the direct determinants of the behavioral intention to use the technology under study, whereas facilitating conditions and behavioral intention to use the technology are the 2 determinants of usage behavior. The UTAUT2, modified from the UTAUT, includes 3 new constructs: hedonic motivation, price value, and habit. In this study, we selected the UTAUT as our theoretical model, as it has been tested more frequently in health care settings [22-24], consequently having higher levels of validation compared with the UTAUT2.

### The Technology: Mobile Smart Care System

**Figure 1:**
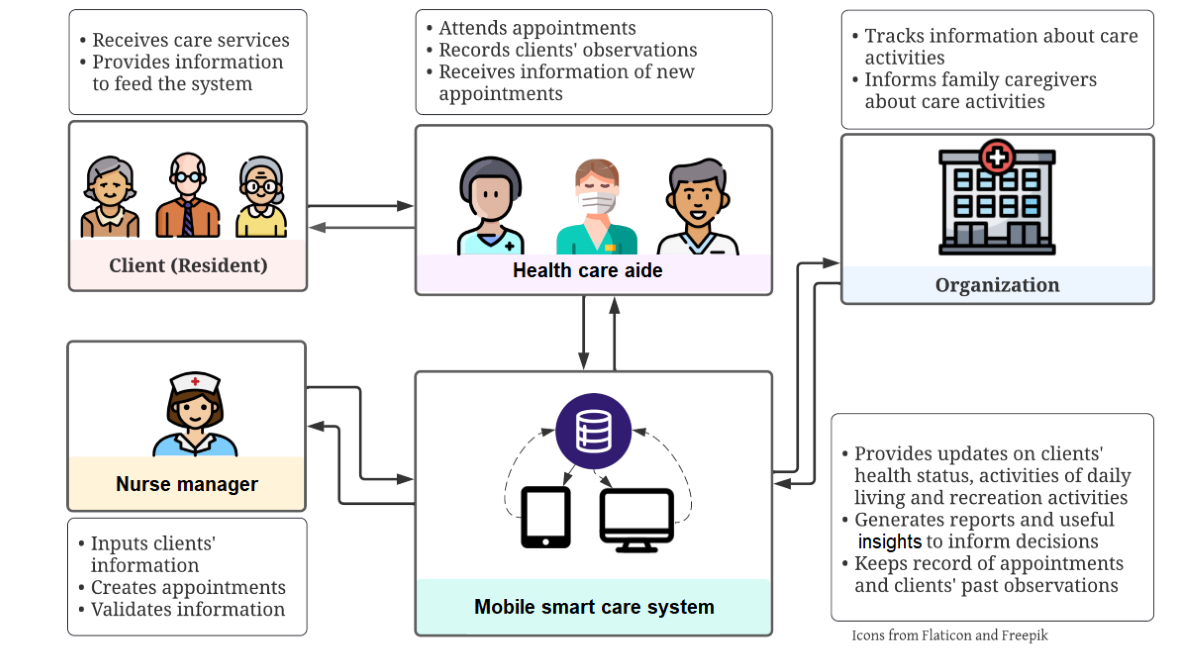
The Mobile Smart Care System architecture at a glance.

Figure 1 shows the architecture of the Mobile Smart Care System (mSCS). The mSCS is a tablet-compatible web-based appl that allows access to an electronic medical record system. The mobile user interface of the mSCS enables health care aides to access their clients’ care plans and observations (eg, bathing, feeding, grooming, dressing, bowel control, bladder control, toilet use, transfer in and out of bed, and mobility) previously uploaded to the electronic medical record by their supervisors (ie, nurse managers). The health care aides recorded their observations and reported their completed activities. The mSCS also enabled supervisors (nurse managers) to monitor health care aides’ care plan activities and observations with an integrated module on the client’s history (previous appointments). The mSCS was installed on tablets using the Android operating system.

## Methods

### Study Design

This was a pilot study using a mixed methods design: sequential mixed methods (*QUANTITATIVE, qualitative*) [25]. Our study included a pre– and post–paper-based questionnaire with no control group (*QUAN*). Toward the end of the study, 2 focus groups were conducted with a subsample of health care aides (*qual*, qualitative description design).

### Setting

This study recruited health care aides from a facility that is part of the Wing Kei Care Centre (Alberta, Canada) from August 17 to October 19, 2021. The Wing Kei Care Centre is home to 145 older adults and has 77 private rooms, 36 semiprivate rooms, and an 80-bed long-term care center. The Wing Kei Care Centre provides culturally specific programs and services for Chinese older adults.

### Sample Size Calculation

The quantitative aspect of the study required a sample size of 60 health care aides to achieve a statistical power of 0.80 with a small effect size (ie, 0.25) and an *α* of .05 for a partial least squares (PLS) structural regression model [26]. The qualitative component of the study involved 10 health care aides. The target sample size was determined based on researchers’ previous experience and existing literature [27].

### Participants

The health care aides were employed at 1 of the 3 sites that are part of the Wing Kei Care Centre. They provided services to older adults as part of their routine caseloads and were recruited using convenience sampling. To be included in this study, the health care aides had to be familiar with using digital technologies such as smartphones or tablets.

### Variables

Intention to use and actual usage behavior related to the mSCS were used as the outcome measures in the multivariate PLS structural regression model (from here on referred to as the PLS model) to determine the factors that had an effect on the acceptance and usage behavior of the mSCS. Performance expectancy, effort expectancy, and social influence were considered direct determinants of behavioral intention with regard to using the mSCS. Behavioral intention regarding the use and facilitation conditions for using the mSCS were treated as direct determinant factors for usage behavior of the mobile app. We included demographic data such as sex, age, level of comfort using digital technologies (eg, computers, smartphones, the internet, and tablets) and years of experience working as a health care aide as potential confounding variables. Dichotomous variables were coded *0* or *1* (eg, sex). Every item in section B-1 in the questionnaire was related to each dependent, and the independent variables were scored on a 5-point Likert scale, ranging from *disagree* (1) to *agree* (5). We calculated 3 summative scores by adding all the items from the UTAUT constructs (except for usage behavior, 10 items), all the items from each UTAUT construct (2 items for each of the 5 constructs), and all the items from the level of comfort using digital technology (4 items). The maximum possible value of the first summative score based on the 5-point Likert scale was 50 points (2 items for each of the 5 constructs). Therefore, a summative score higher than 30 points and closer to 50 points suggests that the technology acceptance of the mSCS was high. The maximum possible value of the second summative score based on a 5-point Likert scale was 10 (2 items per construct). Therefore, a summative score higher than 6 points and closer to 10 points would suggest that performance expectancy, effort expectancy, social influence, facilitating conditions, and behavioral intention to use the mSCS were high. For the third scale, a summative score higher than 12 points and closer to 20 points suggests that health care aides have high levels of comfort in using digital technologies.

### Data Sources and Instruments

Table 1 summarizes the UTAUT constructs using measurement items. We designed and administered a paper-based initial questionnaire (10 items; 2 items per UTAUT construct) and an exit questionnaire (12 items; eg, the exit questionnaire had 2 additional questions about usage behavior with the mSCS) to understand the factors that affected the actual use of the mSCS. The purpose of the initial questionnaire was to obtain a baseline for mSCS acceptance, whereas the exit questionnaire was aimed at understanding usage behavior and whether the health care aides’ expectations of the mSCS were met. The questionnaire for the health care aides had 3 sections. Section A-1 included demographic data such as sex, age, and years of experience working as a health care aide. Section A-2 in the questionnaire used a 5-point Likert scale to determine the health care aides’ level of comfort in using digital technologies, ranging from *disagree* (1) to *agree* (5). Section B-1 included questionnaire items that used a 5-point Likert scale ranging from *disagree* (1) to *agree* (5). The items included in this section were previously validated as having high levels of internal consistency [20,28].

Focus groups were guided by 6 questions that examined the health care aides’ experiences (ie, usefulness and ease of use) with the mSCS during their work day and their satisfaction with the system while carrying out routine tasks. We also asked about the potential influence of the mSCS on the quality of care provided, the challenges and barriers associated with using the system, and the possibility of using the system in a home care setting.

**Table 1:** Summary of the construct and corresponding measurement items.

Construct	Corresponding items (initial questionnaire)	Corresponding items (exit questionnaire)	Source
**PE^a^**			
	PE1: using the mSCS^b^ will improve the management of care for my clients.	PE1: using the mSCS improved my ability to care for my clients.	[20]
	PE2: overall, the mSCS will be useful for doing my job as a health care aide.	PE2: overall, the mSCS was useful for my job.	[20]
**EE^c^**			
	EE1: learning to use the system will be easy for me.	EE1: learning to use the mSCS app was easy for me.	[20]
	EE2: overall, I will find the mSCS easy to use.	EE2: overall, the mSCS was easy to use.	[28]
**SI^d^**			
	SI1: my colleagues at work think that I should use the mSCS to manage my caregiving activities.	SI1: my colleagues think that I should use the mSCS to manage my caregiving activities.	[28]
	SI2: in general, my supervisor will support my use of the mSCS to manage my caregiving activities.	SI2: in general, my supervisor supported my use of the mSCS to manage my caregiving activities.	[28]
**FC^e^**			
	FC1: I will receive good technical support with the mSCS.	FC1: I received good technical support with the mSCS.	[28]
	FC2: the mSCS will be fast to get into.	FC2: the mSCS was fast to get into.	[28]
**BI^f^**			
	BI1: if possible, I will use the mSCS to manage my caregiving activities.	BI1: if it were up to me, I would continue to use the mSCS to manage my caregiving activities.	[28]
	BI2: if possible, I will continue to use the mSCS app to provide a better service to my clients.	BI2: if it were up to me, I would continue to use the mSCS as a way to care for my clients better.	[28]
**UB^g^**			
	N/A^h^	UB1: I used the mSCS to organize my caregiving activities.	[28]
	N/A	UB2: I used the mSCS to manage my caregiving activities.	[28]

^a^PE: performance expectancy.

^b^mSCS: Mobile Smart Care System.

^c^EE: effort expectancy.

^d^SI: social influence.

^e^FC: facilitating conditions.

^f^BI: behavioral intention.

^g^UB: usage behavior.

^h^N/A: not applicable.

### Ethics Approval

This study was approved by the ethics committee of the University of Alberta (Pro00095093).

### Procedures

A member of the research team at the care center sent a letter of invitation along with the information letter and consent form by email to potential participants who matched the inclusion criteria. Health care aides who were interested in participating signed the consent form and then emailed the form back to the project coordinator.

The project coordinator administered the initial questionnaire to each health care aide who agreed to participate in the study. Next, they provided a tablet with the mSCS installed on it to each health care aide and provided instructions on how to use the mSCS. The app also had a tutorial video that taught the health care aides how to use the tablets and access the system through the mSCS (ie, Clinisys portal). Each health care aide used the system for 1 month. After the trial period, the project coordinator emailed each health care aide the exit questionnaire for completion. Health care aides then emailed the completed questionnaires back to the project coordinator. Each health care aide received an honorarium of CAD $25.00 (US $19.99) for each of the research activities completed (ie, the initial usability questionnaire and exit usability questionnaire).

The focus groups were held with health care aides at the care center toward the end of the study. A total of 2 focus groups were conducted, with 6 health care aides in each focus group for 12 health care aides. Thus, the 12 health care aides completed both the quantitative and qualitative components of the study. Each health care aide who participated in a focus group received an honorarium of CAD $25.00 (US $19.99).

### Data Analyses

Descriptive statistics were used to summarize the demographic data of the health care aides. SPSS (version V 28.0; IBM Corp) and SmartPLS (version 3.2.0) [29] statistics packages were used to generate descriptive, univariate, and bivariate statistics and a PLS structural regression model, respectively. The sample size was estimated using G*Power (version 3.1.9.4; Universität Kiel) [30]. Comparisons of the outcome and independent variable summative scores at pretest and posttest were calculated using a paired *t* test. We used a PLS structural regression model to determine the factors that influenced mSCS acceptance and usability for the health care aides. To determine whether to include mediator and moderator variables in the PLS structural model, bivariate correlations (ie, Spearman ρ or Pearson correlation) between performance expectancy, effort expectancy, social influence, behavioral intention to use, and current use of the mSCS that were independent of sex, age, level of comfort using mobile apps, and years of experience working as a health care aide were calculated. Finally, a PLS structural measurement model evaluation was conducted using the following: (1) a reliability measurement for each construct (internal composite reliability [ICR]), (2) a convergent validity measurement of each set of items with respect to their associated construct being assessed by examining the factor loadings of the items on the model’s constructs, and (3) the discriminant validity that was analyzed using an average variance extracted (AVE) indicator. The PLS structural regression model was evaluated using path coefficients, explained variance (*R*^2^), and effect size (*f*^2^) for each path segment of the model. In addition, bootstrapping resampling was used to verify the statistical significance of the path coefficients of the PLS structural regression model. We used 5000 bootstrap subsamples [26]. The alpha level of significance for each test was set at *P*≤.05 (2-tailed).

The focus groups were digitally recorded and transcribed verbatim, using thematic descriptive methods [31]. Thematic analysis guided data analysis. The analyst (ER) began the analysis by inductively generating codes that were refined as the coding progressed. After the coding hierarchy was developed, the key themes were generated. During the analysis, the analyst verified emergent codes and themes through discussion with research team members.

## Results

### Participants

A total of 75 health care aides were invited to participate. Of these, 15 (20%) did not respond to the invitation to schedule the training session and administration of the demographic data form and pretest. In all, 60 (80%) health care aides were enrolled and completed the pretest. A health care aide took part in 2 weeks of the study but dropped out before the exit interview. Thus, the final sample size, with complete initial and exit data, consisted of 59 health care aides. The final number of questionnaires analyzed at the exit phase was for 59 health care aides, representing 98% (59/60) of the cases.

Table 2 shows the demographics of health care aides. Their average age was 45.16 (SD 8.97) years. The health care aides had almost 8 years of work experience (mean 7.43, SD 4.74 years), and almost all were identified as female (59/60, 98%). The health care aides reported high levels of comfort using digital technologies (19.71, SD 1.18).

**Table 2:** Demographics of the health care aides (N=60).

		Values
**Age and work experience, mean (SD)**		
	Age (years)	45.16 (8.97)
	Number of years of experience working as a health care aide	7.43 (4.74)
**Level of comfort using digital technologies, mean (SD)**		
	I am comfortable using a computer	4.95 (0.29)
	I am comfortable using a tablet	4.97 (0.18)
	I am comfortable using a smartphone	4.84 (0.62)
	I am comfortable using the internet	4.95 (0.39)
	Summative scale^a^	19.71 (1.18)
**Gender, n (%)**		
	Female	59 (98)
	Male	1 (2)
	Nonbinary	0 (0)
	Transgender	0 (0)

^a^*Disagree* (1) to *Agree* (5). Summative scale—minimum to maximum: 4 to 25.

### Technology mSCS Acceptance and Usability: Quantitative results and Pre- and Posttest Comparisons

Table 3 shows the descriptive statistics and hypothesis tests (paired *t* tests) of the technology acceptance of the mSCS in terms of a summative scale (all the UTAUT construct items) and for each UTAUT construct, respectively. The results shown in Table 3 indicate that, overall, acceptance of the mSCS was high in the exit interviews, after the health care aides used the mSCS. Overall, the health care aides’ expectations regarding their acceptance of the mSCS were met, as the means of the summative score were >30, and there were no differences between the initial and exit summative scores. These results suggest that the health care aides would continue to use the mSCS in the future if they were able to do so.

**Table 3:** Health care aides’ level of technology acceptance using the Mobile Smart Care System summative scale per Unified Theory of Acceptance and Use of Technology (UTAUT) construct (initial and exit comparisons).

UTAUT constructs	Initial (n=60), mean (SD)	Exit (n=59), mean (SD)	Paired *t* test statistics (2-tailed; n=59)				
			*P* value	*t* test (*df*)	95% CI	Effect size	Power (%)
Performance expectancy	9.37^a^ (1.56)	9.07 (1.92)	.32	1.003 (59)	−0.298 to 0.898	0.221	50
Effort expectancy	9.33^a^ (1.45)	9.43 (1.57)	.72	−0.362 (59)	−0.652 to 0.452	0.090	20
Social influence	9.17^a^ (1.59)	9.02 (1.82)	.61	0.517 (59)	−0.430 to 0.730	0.117	33
Facilitating conditions	9.32^a^ (1.49)	9.42 (1.58)	.72	−0.356 (59)	−0.662 to 0.462	0.090	20
Behavioral intention	9.27^a^ (1.45)	9.23 (1.78)	.90	0.123 (59)	−0.507 to 0.573	0.032	21
Usage behavior	N/A^b^	9.10 (1.96)	N/A	N/A	N/A	N/A	N/A
Summative scale^c^	46.5 (6.96)	46.9 (5.46)	.68	−0.414 (59)	−2.510 to 1.650	0.054	10.9

^a^*Disagree* (1) to *Agree* (5); 2 items per UTAUT construct; minimum summative scale: 2, maximum summative scale: 10.

^b^N/A: not applicable.

^c^Minimum summative scale: 10, maximum summative scale: 50 (all of the Unified Theory of Acceptance and Use of Technology construct items).

Regarding the results for each UTAUT construct, according to health care aides’ responses, they believed the mSCS was useful (high performance expectancy), easy to use (low effort expectancy), fit with their needs (high facilitating conditions), and the influence of others on their use was high. Importantly, health care aides would be willing to use the mSCS in the future if they were able to do so (average intention to use the mSCS, behavioral intention construct 9.27, SD 1.45; maximum 10). At exit, the mSCS showed high levels of usability (average usage behavior with the mSCS, USE [actual use] 9.10, SD 1.96). We did not find any statistically significant differences between the initial and exit summative scores for any of the UTAUT constructs.

### Technology mSCS Acceptance and Usability: Multivariate Analyses (PLS Model)

As we did not find any statistically significant differences between the initial and exit summative scores for any of the UTAUT constructs, we ran only one PLS model (the exit model). The bivariate analysis showed the health care aides’ responses related to performance expectancy, effort expectancy, social influence, behavioral intention, and usage behavior with the mSCS. The constructs were independent of sex, age, level of comfort using digital technologies, and years of experience working as health care aides (Multimedia Appendix 1).

The PLS results for the structural model are shown in Table 4. During the exit interview, we found, as the UTAUT model predicted, a strong positive correlation between usefulness (performance expectancy; performance expectancy→behavioral intention, β=.856; *P*=.004) and behavioral intention to use the mSCS. However, contrary to what the UTAUT suggests, we found that effort expectancy (degree of ease of use; effort expectancy→behavioral intention, β=−0.083; *P*=.57) and social influence (social influence→behavioral intention, β=.044; *P*=.83) were not salient constructs for intention to use the mSCS. In addition, as the UTAUT model predicted, we found a strong positive and statistically significant correlation between behavioral intention to use the mSCS and usage behavior with the mSCS (behavioral intention→usage behavior, β=.789; *P*<.001). Finally, we also found that although the facilitating conditions and usage behavior were positively correlated, as predicted by the UTAUT model (ie, facilitating conditions→ usage behavior, β=.098; *P*=.47); this relationship was not statistically significant in this study.

**Table 4:** Determinants of behavioral intention and usage behavior regarding the Mobile Smart Care System (5000 bootstrap subsamples).

Path segment	Health care aides (n=59)							
	β^a^	*t* test statistics (df=59)	*P* value	95% CI	*f* ^2b^	*R* ^2c^	*R* ^c^_adjusted_	Power %
PE^d^ →BI^e^	0.856	2.906	.004	0.029 to 1.154	0.689	0.690	0.673	100
EE^f^→BI	−0.083	0.566	.57	−0.313 to 0.266	0.010	—^g^	—	—
SI^h^→BI	0.044	0.214	.83	−0.201 to 0.600	0.002	—	—	—
BI→UB^i^	0.789	7.672	<.001	0.559 to 0.976	1.474	0.748	0.739	100
FC^j^→UB	0.098	0.716	.47	−0.146 to 0.388	0.022	—	—	—

^a^Path coefficients.

^b^Effect size.

^c^Explained variance.

^d^PE: performance expectancy.

^e^BI: behavioral intention.

^f^EE: effort expectancy.

^g^R2 (R^c^_adjusted_) and power are calculated for constructs BI (PE, EE, and SI contributes to the explained variance of BI) and UB (BI and FC contribute to the explained variance of UB).

^h^SI: social influence.

^i^UB: usage behavior.

^j^FC: facilitating conditions.

### PLS Model Validity and Reliability

Table 5 shows the results of the construct correlations and descriptive statistics, ICR, Cronbach α, and AVE of the constructs of the PLS. The square root of each AVE (shown on the diagonal in Table 5) was greater than the related interconstruct correlations in the construct correlation matrix, indicating adequate discriminant validity for all the constructs. All AVE values were >0.5, indicating good convergent validity at the construct level [26]. All ICR and Cronbach α values were >.70, indicating good internal consistency at the construct level [26]. The PLS models also showed that all item loadings were statistically significant at the 0.001 level, and 100% of the item loadings were >0.70, indicating excellent values of convergent validity at the indicator level [26] (see Table 6 for more details). The explained variance (ie, *R*^2^) of the constructs of the PLS model was 0.690 and 0.748 for behavioral intention to use the mSCS and actual usage behavior with the mSCS, respectively, which appears to be strong according to the published criteria [26].

**Table 5:** Construct correlations and construct reliability and validity of the partial least squares structural regression model (n=59).

Construct	Values, mean^a^ (SD)	ICR^b^	Cronbach α	AVE^c^	BI^d^	EE^e^	FC^f^	PE^g^	SI^h^	UB^i^
BI	9.23 (1.78)	0.972	.943	0.946	0.973^j^	—^k^	—	—	—	—
EE	9.43 (1.5)	0.885	.741	0.794	0.569^l^	0.891^j^	—	—	—	—
FC	9.41 (1.57)	0.883	.756	0.791	0.646^l^	0.899^l^	0.890^j^	—	—	—
PE	9.06 (1.92)	0.965	.928	0.932	0.828^l^	0.731^l^	0.792^l^	0.966^j^	—	—
SI	9.01 (1.82)	0.916	.821	0.845	0.655^l^	0.605^l^	0.606^l^	0.774^l^	0.919^j^	—
UB	9.10 (1.96)	1.000	1.000	1.000	0.562^l^	0.562^l^	0.614^l^	0.806^l^	0.708^l^	1.00^j^

^a^*Disagree* (1) to *Agree* (5); 2 items per Unified Theory of Acceptance and Use of Technology construct; minimum summative scale: 2, maximum summative scale: 10.

^b^ICR: internal composite reliability.

^c^AVE: average variance extracted.

^d^BI: behavioral intention.

^e^EE: effort expectancy.

^f^FC: facilitating conditions.

^g^PE: performance expectancy.

^h^SI: social influence.

^i^UB: usage behavior.

^j^Square root of AVEs reported along diagonal (Fornell-Larcker criterion).

^k^—: not applicable.

^l^*P*<.01.

**Table 6:** Reliability and convergent validity of the partial least squares structural regression model—measurement model (n=59).

Construct and item		Item loading	*t* test (df=59)	95% CI	ICR^a^		AVE^b^		Cronbach α	
**PE^c^**						0.965		0.932		.928
	PE1	0.965	35.699^d^	0.884 to 0.987						
	PE2	0.966	48.068^d^	0.909 to 0.988						
**EE^e^**						0.885		0.794		.741
	EE1	0.884	6.300^d^	0.446 to 0.965						
	EE2	0.899	13.774^d^	0.828 to 1.000						
**SI^f^**						0.916		0.845		.821
	SI1	0.947	41.169^d^	0.891 to 0.984						
	SI2	0.897	12.970^d^	0.693 to 0.954						
**FC^g^**						0.883		0.791		.756
	FC1	0.950	24.337^d^	0.896 to 0.993						
	FC2	0.824	6.810^d^	0.485 to 0.953						
**BI^h^**						0.972		0.946		.943
	BI1	0.972	32.278^d^	0.900 to 0.993						
	BI2	0.973	46.060^d^	0.909 to 0.993						
**UB^i^**						1.000		1.000		1.000
	UB1	Deleted	N/A^j^	N/A						
	UB2	1.000	N/A	N/A						

^a^ICR: internal composite reliability.

^b^AVE: average variance extracted.

^c^PE: performance expectancy.

^d^*P*<.01.

^e^EE: effort expectancy.

^f^SI: social influence.

^g^FC: facilitating conditions.

^h^BI: behavioral intention.

^i^UB: usage behavior.

^j^N/A: not applicable.

### Technology MSCS Acceptance and Usability: Qualitative Results

#### Focus Groups

Of 59 health care aides who completed the exit questionnaire, 12 (20%) participated in the focus group. The health care aides’ average age was 46.83 (SD 7.5) years, they had almost 9 years of work experience (mean 9.01, SD 4.08 years), and almost all identified as female (11/12, 92%). The following themes emerged from the qualitative data analysis: (1) the mSCS is useful, (2) the mSCS is portable and reliable, and (3) there are still opportunities for improvement.

##### The mSCS Is Useful

Most health care aides valued the usefulness and relative advantage of the mSCS in their daily lives, compared with not using an electronic system to access care plans and tasks. Health care aids identified that the mSCS saved time, “because we can document right away whatever we observed the resident, rather than paperwork and it takes time” (participant HCA1P2). They also stated that using the system is “faster than handwriting,” and “prevents spelling errors” (participant HCA1P6). Using the mSCS on a tablet was convenient and allowed for multitasking, as described in the statement by participant HCA2P1 “[you] can just bring this along [the tablet] with the resident when we’re waiting for them.” The mSCS was informative when health care aides completed care tasks. For example, according to participant HCA1P2, “If residents, are new, I can check in the system [mSCS]. I can check the care plan and about their status. So I don’t even ask my co-worker…” Having a resident’s medical history that was easily accessible was useful for quick reference. This was especially true for new staff, such as participant HCA2P3, who stated, “If we have new clients, we have the reporting time [that] shares the information for the new client.”

##### The mSCS Is Portable and Reliable

Most health care aides valued having the mSCS on a tablet, as it made it portable and reliable. Regarding portability, participant HCA2P2 commented, “It’s very handy, you can carry it anywhere.” Some health care aides also believed that having the mSCS on a tablet allowed better accessibility to the “system” in comparison with having the information in a point-of-care system located on a computer at the nurse’s station. By reliability, the participants meant that they only needed to wait for a short time for connection to save their data. For example, participant HCA2P3 mentioned “And the system won’t hang up. You can just go straight forward. In between it won’t break down...”

##### There Are Still Opportunities for Improvement

Most health care aides mentioned that the mSCS user interface was easy to understand and enter information; however, many of its aspects needed to be improved. For example, participant HCA1P3 stated that they faced some “interface issues”:

…the little dots to input the selection [are a very small interface]. It probably should be a bigger block so you can easily click it because sometimes when you’re clicking it clicks [and you go] onto the different [places]… [and it causes] a wrong selection…

Participant HCA2P3 stated that the font size needed to be enlarged “because we are aging.” The health care aides mentioned that visual indicators or aids, such as color, could help to quickly and easily confirm that information was entered correctly. The lack of these interface elements led to incorrect documentation and sometimes made the mSCS confusing to use. For example, HCA2P4 stated the following:

There’s lots of dates. And we’ve been confused with those dates because, oh, I haven’t started this thing, how come the date is here?

Finally, although the health care aides appreciated the simplicity of the fields when they were entering information, an option to include more detailed information was strongly recommended.

## Discussion

### Principal Findings

In this study, we aimed to investigate the technology acceptance and usability of a mobile app in a real-world environment used by health care aides who provide services to older adults. We found that (1) the acceptance of the mSCS was high among health care aides, (2) the performance expectancy construct was the only predictor of intention to use the mSCS, and (3) the intention to use the mSCS predicted usage behavior with the mSCS. The qualitative data supported the quantitative findings, showing the health care aides’ strong belief that the mSCS was useful, portable, and reliable; although, they also suggested opportunities for improvement, especially in the mSCS user interface. Overall, these results support the assertion that mSCS technology acceptance and usability were high among health care aides.

We found that the performance expectancy construct was a predictor of intention to use the mSCS. In other words, the health care aides accepted the mSCS because it improved their ability to care for their clients. This finding is consistent with the results of previous studies on the application of UTAUT in mHealth, health, rehabilitation, and assistive technologies [28,32,33]. Lim et al [32] found that performance expectancy had a significant influence on primary care physicians’ acceptance of mHealth technology (ie, use of mHealth apps to support their clinical work). Liu et al [28] revealed that performance expectancy had a significant influence on the acceptance of GPS technology among people living with dementia and family caregivers. Finally, Liu et al [33] reported that performance expectancy was the most significant factor in new technologies for rehabilitation acceptance.

Health care aides had the perception that mSCS assisted their workflows. Qualitative and quantitative analyses showed that the perception of usefulness and relative advantage of the mSCS in health care aides daily work, and the workflow was superior compared with not using an electronic system. The usefulness and relative advantage of the mSCS are related to documentation and charting tasks. In other words, as the health care aides believed that the mSCS saved time with documentation and charting tasks, resulting in more time to provide care to older adults.

Effort expectancy was not a factor affecting intention to use the mSCS. In fact, although not statistically significant, we obtained a negative correlation between effort expectancy and intention to use the mSCS. This meant that the health care aides perceived some issues regarding mSCS use, although they would continue to use this technology if they are able to do so. This result is consistent with that of previous studies [33-36]. Liu et al [33] found that effort expectancy was not a significant factor in the acceptance of new technologies for rehabilitation. A meta-analysis conducted by Taiwo and Downe [34] reported that the effects of effort expectancy on intention to adopt were weak or had no significance. Braun [35] found support for the premise that users’ effort expectancy partially predicted their intention to use social networking websites [35]. As far as our study is concerned, the fact that effort expectancy was not a factor in the intention to use the mSCS may have different explanations. The most plausible reason for this is that the mSCS user interface still requires improvement. Comments from the health care aides during the focus groups revealed that they “experienced difficulties completing the report due to a lack of options [in the user interface],” and sometimes they encountered “interface issues.”

In this study, social influence was not a factor affecting intention to use the mSCS. In other words, the health care aides were not influenced by the degree of difficulty or social pressure from their colleagues or supervisors toward using the mSCS. As is evident in the literature, the role of social influence on behavioral intention to use technologies has mixed results. In some studies, social influence was a factor that affected the intention to use the technologies under study [28], whereas in other studies, this was not the case [23,33]. The meta-analysis conducted by Taiwo and Downe [34] revealed small effect sizes for social influence, showing conflicting evidence that social influence is salient for technology acceptance. Future research on technology acceptance should continue to explore whether social influence affects intention to use technologies.

The combined results of performance expectancy, effort expectancy, and social influence on intention to use the mSCS suggest the following: in a nonmandatory health care setting, no matter how difficult it is to use the mSCS, or whether there is external social pressure to use the mSCS, health care aides will only accept the mSCS if they perceive it will help them attain their goals at work.

We found that facilitating conditions did not affect usage behavior in the mSCS. This finding was surprising, as previous studies in the areas of eHealth, mHealth, and assistive and rehabilitation technologies have consistently found the opposite [23,28,33,37,38]. One possible reason we obtained this result is because the technology under study was used under *ideal* conditions, that is, we had a dedicated project coordinator who served as an *intermediary* between the health care aides and the technology provider (ie, Clinisys EMR Inc), which meant that issues with use were immediately resolved, and we had a dedicated nurse manager who inputted older adults’ information into the mSCS. These 2 conditions may not have allowed the health care aides to experience the importance of having good technical support, as they did not have to interact with the service provider.

Finally, we found that intention to use predicted usage behavior with the mSCS. This result supports the core tenets of the UTAUT model, that is, if health care aides have the intention to use the technology (ie, mSCS), they will use it if they are able to do so. In more concrete terms, the mSCS was accepted, and as a result, it would be adopted by health care aides.

### Limitations

This study has 5 limitations. First, as this study was conducted in only one long-term care facility, it represents a starting point for investigating the crucial factors that influence health care aides’ intention to adopt a mobile app and usage behavior of a mobile app. Consequently, we caution against generalizing our results to other health service providers as well as other long-term care facilities. Second, all but one of our participants were identified as female (59/60, 98%). In the future, it would be ideal to have an equal number of men and women represented in the data analyses to examine gender differences. Third, the health care aides who returned the technology acceptance and usability questionnaire might have been inclined to prefer the mSCS and, thus, were willing to fill out the questionnaire. Fourth, the results of our pre- and posttest for our variables resulted in a statistical power that was lower than the conventional cutoff value of 0.80. Thus, future studies should pursue larger sample sizes when the effect size is low. Fifth, we did not record the cultural or language demographic characteristics of the health care aides; as a result, we are unable to assert whether cultural or language factors affect the technology acceptance and usability of the mSCS for this population. Finally, we experienced a ceiling effect (ie, most of the values obtained for our constructs approached the upper limit of the technology acceptance and usability questionnaire). Thus, in future studies, it would be reasonable to use a 7-point Likert scale in technology acceptance and usability studies, especially when the scale is new.

### Conclusions

This study clearly showed that mSCS was accepted by the health care aides. The study also surpassed expectations regarding the technological acceptance of the mSCS, which were found to have been met for all the health care aides. In conclusion, the results suggest that health care aides would continue to use the mSCS if they were able to do so. The health care aides found the mSCS to be useful, portable, and reliable. They perceived that mSCS addressed the workflow issues.

## Appendix

Multimedia Appendix 1 Correlational analyses of confounding variables.

## Abbreviations

AVE: average variance extracted
ICR: internal composite reliability
ICT: information communication technologies
mHealth: mobile health
mSCS: Mobile Smart Care System
PLS: partial least squares
UTAUT: Unified Theory of Acceptance and Use of Technology
