# Supplementary material for: Technology Acceptance and Usability of a Mobile App to Support the Workflow of Health Care Aides Who Provide Services to Older Adults: Pilot Mixed Methods Study
Source: JMIR Aging. 2022 May 18;5(2):e37521. doi: 10.2196/37521 (PMC9161048; doi:10.2196/37521)
Supplement: Multimedia Appendix 1 [file aging_v5i2e37521_app1.docx]

| **Variable name** | **1** | **2** | **3** | **4** | **5** | **6** | **7** | **8** | **9** | **10** |
| --- | --- | --- | --- | --- | --- | --- | --- | --- | --- | --- |
| 1. Gender | 1 |  |  |  |  |  |  |  |  |  |
| 1. Lebel of Conf | -0.041 | 1 |  |  |  |  |  |  |  |  |
| 1. Years of experience | 0.067 | -0.042 | 1 |  |  |  |  |  |  |  |
| 1. Age | 0.198 | -0.260 | 0.634** | 1 |  |  |  |  |  |  |
| 1. PE | 0.073 | 0.011 | -0.094 | 0.027 | 1 |  |  |  |  |  |
| 1. EE | 0.120 | 0.043 | -0.075 | 0.048 | 0.841** | 1 |  |  |  |  |
| 1. SI | 0.073 | 0.009 | 0.035 | 0.116 | 0.864** | 0.795** | 1 |  |  |  |
| 1. FC | -0.049 | 0.076 | -0.062 | 0.024 | 0.853** | 0.962** | 0.796** | 1 |  |  |
| 1. BI | 0.017 | 0.028 | -0.072 | 0.099 | 0.898** | 0.790** | 0.806** | 0.829** | 1 |  |
| 1. USE | -0.060 | 0.034 | -0.100 | 0.072 | 0.879** | 0.746** | 0.821** | 0.781** | 0.915** | 1 |
| **. Correlation is significant at the 0.01 level (2-tailed). | | | | | | | | | | |

*Supplemental material.*

Table S1. Correlational analysis. Confounding variables
